# Novel Antioxidant Insights of Myricetin on the Performance of Broiler Chickens and Alleviating Experimental Infection with *Eimeria* spp.: Crosstalk between Oxidative Stress and Inflammation

**DOI:** 10.3390/antiox12051026

**Published:** 2023-04-28

**Authors:** Waleed Rizk El-Ghareeb, Asmaa T. Y. Kishawy, Reham G. A. Anter, Asmaa Aboelabbas Gouda, Walaa S. Abdelaziz, Bassam Alhawas, Ahmed M. A. Meligy, Sherief M. Abdel-Raheem, Hesham Ismail, Doaa Ibrahim

**Affiliations:** 1 Department of Public Health, College of Veterinary Medicine, King Faisal University, P.O. Box 400, Hofuf 31982, Al-Ahsa, Saudi Arabia; 2 Food Control Department, Faculty of Veterinary Medicine, Zagazig University, Zagazig 44519, Egypt; 3 Department of Nutrition and Clinical Nutrition, Faculty of Veterinary Medicine, Zagazig University, Zagazig 44511, Egypt; 4 Department of Parasitology, Faculty of Veterinary Medicine, Zagazig University, Zagazig 44511, Egypt; 5 Avian and Rabbit Medicine Department, Faculty of Veterinary Medicine, Zagazig University, Zagazig 44511, Egypt; 6 Department of Clinical Science, Central Lab, College of Veterinary Medicine, King Faisal University, P.O. Box 400, Hofuf 31982, Al-Ahsa, Saudi Arabia; 7 Department of Physiology, Agricultural Research Center (ARC), Giza 12511, Egypt; 8 Department of Animal Nutrition and Clinical Nutrition, Faculty of Veterinary Medicine, Assiut University, Assiut 71526, Egypt; 9 Food Hygiene Department, Faculty of Veterinary Medicine, Assiut University, Assiut 71526, Egypt

**Keywords:** myricetin, flavonoid, performance, antioxidant, inflammation, *Eimeria* spp.

## Abstract

In the modern poultry industry, the application of novel phytogenic bioactive compounds with antioxidant potential aims to enhance productivity and quality and to minimize the stress of associated diseases. Herein, myricetin, a natural flavonoid, was evaluated for the first time on broiler chickens’ performance, antioxidants and immune modulating functions, and tackling avian coccidiosis. A total of 500 one-day-old chicks were divided into five groups. The negative (NC) and infected control (IC) groups were fed a control diet without additives, and the latter was infected with *Eimeria* spp. Groups supplemented with myricetin (Myc) were fed a control diet of Myc (200, 400 and 600 mg/kg diet each). On d 14, all chicks except those in NC were challenged with oocysts of mixed *Eimeria* spp. Significant improvements in the overall growth rate and feed conversion ratio were detected in the group that was fed 600 mg/kg, unlike the IC group. Notably, groups that were fed 400 and 600 mg/kg showed higher total meat antioxidant capacity with an inverse reduction in oxidative and lipid peroxidation biomarkers (hydrogen peroxide: H_2_O_2_; reactive oxygen species: ROS; Malondialdehyde: MDA). Of note, the upregulation of glutathione peroxidase; *GSH-Px*, catalase; *CAT*, superoxide dismutase; *SOD*, heme oxygenase-1; *HO-1* and NAD(P)H dehydrogenase quinone 1 *NQO1* genes in jejunum and muscle were prominently observed with increasing levels of supplemental Myc. At 21 dpi, the severity of coccoidal lesions (*p* < 0.05) induced by mixed *Eimeria* spp. and oocyst excretion were greatly reduced in the group that was fed 600 mg/kg of Myc. In the IC group, higher serum levels of C-reactive protein; CRP and nitric oxide; and NO and the upregulated expression of inflammatory biomarkers (interleukin-1β; IL-1β, interleukin-6; IL-6, tumor necrosis factor-α; TNF-α, chemotactic cytokines; CCL20, stromal cell-derived factor-1; CXCL13, and avian defensins; AvBD612) were subsided in higher levels in the Myc-fed groups. Taken together, these findings indicate the promising antioxidant role of Myc in modulating immune responses and reducing growth depression associated with coccidia challenges.

## 1. Introduction

The poultry gastrointestinal tract (GIT) offers a biological environment for nutrient digestion and absorption, as well as protection from pathogens and toxins. Oxidative stress in birds’ GIT is derived from nutritional factors, environmental heat stress, and pathological factors, which alter the overall performance as well as meat quality [1,2]. Biological damage associated with oxidative stress can cause many degenerative health issues, which have a great impact on the overall performance and productivity of livestock [3]. Studies have suggested that the interaction of mucosa with microbes or their toxins triggers oxidative stress [4,5,6]. The supplementation of antioxidant-rich diets and plant extracts having antioxidant properties that scavenge reactive oxygen species (ROS) are beneficial in mitigating oxidative stress in the GITs of animals [7,8,9]. Avian coccidiosis is among the most common parasitic diseases caused by genus *Eimeria* spp. in the poultry industry that is responsible for great economic losses, increasing mortality and lowering growth rates [10]. *Eimeria* spp., primarily producing proinflammatory mediators together with oxidative stress, contributes to lipid peroxidation, antioxidant insults, damage of the intestinal epithelial barrier, inflammatory injury and diarrhea [11]. Following infection with parasites, particularly with *Eimeria* spp., the antioxidant systems of chickens are significantly disrupted. The use of antioxidants is critical in combating the oxidative stressors caused by the production of ROS and for the maintenance of homeostasis [12]. Under the conditions of ROS overproduction, supplementation with compounds of high antioxidant potential is immensely valuable [13,14]. Concomitantly, regarding the prolonged use of common traditional approaches, such as coccidacidal pharmaceuticals that are used for controlling avian coccidiosis, these chemotherapeutics have been banned due to decreased *Eimeria* spp. sensitivity and developed drug resistance [15,16]. Therefore, the dietary inclusion of bioactive compounds that have new antioxidant and immunological prophylactic properties can solve these previous issues by exerting specific coccidiastat effects [17,18]. Prior studies have recommended that natural phytogenic-derived agents, such as resveratrol, oregano essential oil and aloe vera, have profound impacts on ameliorating oxidative stress for animals [19,20]. Moreover, phytogenic substances, rich in isoflavones, have been recognized as alternative additives to replace antibiotic usage in poultry farming via enhancing intestinal integrity and controlling inflammatory signaling pathways [21]. The anticoccidial properties of several natural herbal products (or their extracts) is mainly attributed to their ability to lower the impact on the output of oocyst via the inhibition or suppression of the invasion, replication and expansion of *Eimeria* ssp. in chickens’ gastrointestinal tissues [22].

Moreover, the therapeutic potential and defensive role of these phytogenics against artificially induced coccidiosis in chickens is mainly due to their phenolic compounds that interact with cytoplasmic membranes, triggering coccidial sporozoite death, attenuating intestinal lipid peroxidation, facilitating epithelial injury repair and lessening the intestinal permeability triggered by *Eimeria* spp. [23]. Phenolic compounds, such as flavones (apigenin and luteolin), hydroxycinnamic, caffeic and sinapic acids, have been tested for their inhibitory effective role on the sporulation of coccidian oocysts [24,25,26]. Thus, searching for a new bioactive phenolics candidate with anticoccidial efficacy is an important prerequisite. Myricetin is a natural flavonoid with known strong antioxidant, anti-inflammatory and anticancer properties [27,28]. Additionally, the antiparasitic effect of myricetin was recently documented against Schistosomiasis [29]. The potential antioxidant activity of myricetin could be related to the presence of three hydroxyl groups on its B ring like other flavonoids [30]. Furthermore, myricetin has also proved to modify inflammatory diseases by suppressing pro-inflammatory mediators (inhibition of inflammatory mediators such as TNF-α, IL-6, IL-12 and iNOS) [31,32]. Myricetin can effectively reduce oxidative stress via down-stream expression of HO-1 and NQ1 [33]. At present, the impact of myricetin on combating oxidative stress and avian coccidiosis has not been reported yet in the poultry industry. Hence, this study was designed to assess the effective role of in-feed myricetin on broiler chickens’ performance indices, antioxidant and immune modulation, fecal oocyst excretion and intestinal lesion score following *Eimeria* ssp. challenges.

## 2. Materials and Methods

Husbandry, ethics and guidelines for animal management practices and techniques were obtained from the Institutional Animal Care and Use Committee of the Faculty of Veterinary Medicine at Zagazig University (ZU-IACUC/2/F/320/2020).

### 2.1. Birds, Experimental Design and Growth Monitoring

Five hundred one-day-old male chicks with an initial body weight of 44.2 ± 0.2 g were delivered by a local hatchery (commercial Ross 308 broiler chicks). Chicks were weighed and randomly divided into five equal experimental groups, with 5 replicate pens of 10 birds each. All birds were housed in floor pens with wood shavings (bird density: 10 broilers/m^2^) in similar environmental and sanitary circumstances throughout the experimental time. The room temperature throughout the 1st week was adjusted to be 33 °C and then progressively declined until reaching 23 °C. Three diets were offered during the trial in a crumble form: starter (1 to 10 d), grower (11 to 20 d) and finisher (21 to 42 d) diets, which were formulated to furnish the Ross broilers’ nutrient requirements in accordance with the nutritional specification of ROSS [34]. All birds were fed and watered with ad libitum access. Experimental groups were established as follows. There were two control groups. NC birds received a basal diet without additives and were not challenged. IC birds received a basal diet without additives and were challenged at d 14 of age with *Eimeria* spp. Birds in groups 2, 3 and 4 received a basal diet supplemented with myricetin (Myc, sigma Aldrich, Product No, 529-44-2) at concentrations of 200 (Myc 200), 400 (Myc 400) and 600 (Myc 600) mg/kg diet, respectively, and were challenged at d 14 of age with *Eimeria* spp. The NC group was isolated in a separate area and was checked to be free from coccidian infection via fecal examination at d 14 and 21 of the experiment.

The levels of feed ingredient and chemical composition of the control diet are listed in Table 1, formulated according to the Ross broiler guidelines [35]. The proximate analysis of the feed components was made along with the standard procedures of the Association of Official Agricultural Chemists [36].

### 2.2. Experimental Challenge by Eimeria spp.

A potassium dichromate solution (2%) was used for the induction of oocysts sporulation, followed by washing numerous times with tap water for potassium dichromate removal. At 14 days of age, experimental birds were challenged with *Eimeria* spp. via gavaging a 2 mL suspension of sporulated oocysts from *E. tenella* (5.0 × 10^3^), *E. maxima* (7.0 × 10^3^) and *E. acervulina* (3.5 × 10^4^) into the crop via a plastic syringe that fitted with a plastic cannula. Before gavaging the birds with sporulated coccidian oocysts, their number per mL was checked via microscope utilizing a McMaster counting chamber.

### 2.3. Growth Performance Parameters

The initial individual weights of the chicks were determined on the day of arrival, and then body weight (BW) and the average daily feed intake (FI) in each replicate pen were recorded to calculate the body weight gain (BWG) and feed conversion ratio (FCR) for the entire experimental period (d 1–42), as previously reported by Kishawy et al. [37]. Mortality was noted daily and then calculated for each at the end of the experiment as a percentage of the total bird number.

### 2.4. Fecal Oocytes Shedding of Eimeria spp.

For oocyst output determination, at 7-, 14- and 21-days post-infection (dpi), the total fecal output of each replicate pen was weighed, and fecal samples were collected daily, homogenized thoroughly, and directly examined for *Eimeria* spp. oocytes. One gram of a homogenized fecal sample was diluted 10-fold first with tap water and was then diluted with a saturated saline solution (1:10), and finally, the oocyst counts were determined according to [38] and expressed as the number of oocytes for each g of feces.

### 2.5. Intestinal Lesion Score

Five birds from each group were euthanized and slaughtered via cervical dislocation at 7 dpi for the determination of the intestinal lesion score. The intestines were directly removed and segmented into the duodenum, jejunum, ileum and cecum, and then each segment was opened. The lesion scores were performed according to [39] and ranged from 0 (no gross lesion) to 4 (most severe gross lesion), as follows: 0 refers to a normal intestinal sigment with no observed lesions, 1 refers to a small scattered petechiae, 2 refers to numerous petechiae, 3 refers to extensive hemorrhaging, and 4 refers to extensive hemorrhaging that causes dark colors in the intestinal sigment.

### 2.6. Biochemical Measurements

Blood samples were aseptically collected from birds’ wing veins (one bird/replicate) for immunological analysis. Blood samples were centrifuged for 10 min at 3000 rpm for serum separation. At 4 days pre-infection and at 14 and 21 dpi post *Eimeria* spp. challenge, a clear serum was used for the assessment of myeloperoxidase (MPO), nitric oxide (NO) and C-reactive protein (CRP) activities utilizing commercial kits (Jiancheng Biotechnology Institute, Nanjing, China). Serum immunoglobulin G (IgG) levels were quantified via an enzyme-linked immunosorbent assay (*ELISA*) as previously described [40].

### 2.7. Oxidative and Antioxidant Evaluation

At the end of the experimental period (42 days), intestinal tissue samples and breast meat were collected and thoroughly homogenized for evaluating the oxidative and antioxidant biomarkers. Lipid oxidation measurements were assessed via a thiobarbituric acid-reactive assay (TBARS) value, as defined by Ahn, Olson [41], and TBARS values were expressed as nmol/g of tissue. The total antioxidant capacity (T-AOC) was determined using a commercial assay kit (Sigma-Aldrich, MAK187). Hydrogen peroxide (H_2_O_2_) levels were assessed according to Loreto and Velikova [42], and their levels were expressed as μmoL/g of tissue. Moreover, reactive oxygen species (ROS) were assessed in accordance with the method of LeBel, Ischiropoulos [43].

### 2.8. Gene Expression by Reverse Transcription Quantitative Real-Time PCR (RT-qPCR)

Intestinal tissues and breast meat samples were collected at the end of the experiment (42 days of age) for examining the expression levels of genes encoding glutathione peroxidase (*GSH-Px)*, super oxide dismutase (*SOD*), catalase, NAD(P)H dehydrogenase quinone 1 (*NQO1*) and heme oxygenase-1 (*HO-1*), cyclooxygenase-2 (*COX-2*),. interleukin (IL)-6, *IL-1β* and *IL-10*, tumor necrosis factor-α (*TNF*-α), (chemokine C–C motif ligand 4, also known as macrophage inflammatory proteins-1β), (*CCL4*), chemokine C–C motif ligand 20, also known as macrophage inflammatory proteins-3 α (*CCL20*) and stromal cell-derived factor-1 (*CXCL13*) Avian β-defensin 6 and 12 (*AvBD6* and *AvBD612*). The isolation of total RNA was performed utilizing QIAamp RNeasy Mini kit (Qiagen, Hilden, Germany), and the RNA concentration was quantified at 260 nm via a spectrophotometer. The assay of one-step RT-qPCR was achieved on the Stratagene MX3005P real-time PCR using a QuantiTect SYBR Green RT-PCR Kit (Qiagen, Hilden, Germany). All measurements of PCR were employed in triplicate. The distinction of each PCR amplification assay was validated via an analysis of the final melting curve. Various transcript levels were then standardized by using glyceraldehyde 3-phosphate dehydrogenase (GAPDH) as an endogenous control. All gene-distinct primer sequences employed in RT-qPCR assay are listed in Table 2. The results of the relative mRNA expression of the investigated genes were evaluated using the 2^−ΔΔCt^ method [44].

### 2.9. Statistical Analysis

Statistical data analysis was achieved via the general linear method (GLM) of SPSS. The homogeneity among experimental groups was estimated using Levene’s test, and normality was assessed using the Shapiro–Wilk test. Tukey’s test was performed to distinguish the mean values and variations that were significant. Data variation was conveyed as the standard error of the mean (SEM), and the statistical significance was adjusted at a *p* value of less than 0.05. All graphs were created by GraphPad Prism software Version 8.

## 3. Results

### 3.1. Growth Performance

The impact of supplementing diets with varying levels of Myc on the growth performance parameters in response to *Eimeria* spp. infection is shown in Table 3. At the end of the starter period, the BW and BWG were significantly (*p* < 0.05) improved in groups supplemented with Myc in a dose-dependent manner compared to the control groups. Feed intake was also significantly (*p* < 0.05) increased in the Myc-supplemented groups compared to the control groups. A superior feed conversion ratio was found (*p* > 0.05) in the group that was fed the 600 mg/kg diet of Myc. Regarding the grower period, the most lowered BW and BWG was (*p* < 0.05) detected in the IC group. Moreover, Myc supplementation at higher levels restored the BW and BWG nearly the same as that in the NC group. Higher feed intake was recorded for the IC group, and then it decreased (*p* < 0.05) gradually with Myc inclusion in a dose-dependent manner. The best FCR was noted for the NC group, and the worst one was recorded for the IC group. Feeding with Myc improved (*p* < 0.05) FCR when compared with the IC group in a dose-dependent manner. The performance parameters at the end of the finisher period were nearly the same as that of the grower period, as BW and BWG were significantly (*p* > 0.05) decreased in the IC group compared with the NC group and then significantly (*p* > 0.05) increased with higher levels of Myc supplementation (400 and 600 mg/kg diet). The lowest feed intake during the finisher period (*p* < 0.05)) was found in the IC and Myc200 groups, and the NC group exhibited the highest significant (*p* < 0.05) feed intake. Moreover, FCR was significantly (*p* > 0.05) increased in the IC group, and the NC, Myc 400 and Myc 600 supplemented groups did not significantly (*p* > 0.05) differ. Regarding the overall performance parameters, inferior (*p* < 0.05) performance parameters were noticed for the IC group, and Myc supplementation improved all these parameters. Notably, no significant difference (*p* > 0.05) in FCR was detected between the infected group fed with Myc 600 and the non-infected group fed with the basal diet.

### 3.2. Oxidative and Antioxidant Status in Muscle and Intestinal Tissues

As displayed in Table 4, significant higher T-AOC levels (*p* < 0.05) were detected with increasing the supplemental level of Myc in both intestinal and muscle tissues. A noticeable reduction (*p* < 0.05) in lipid peroxidation biomarkers (MDA) was found in groups fed with Myc-supplemented diets in a dose-dependent manner in intestinal and muscle tissues. A remarkable decline (*p* < 0.05) in ROS production was detected in the muscle tissues of the group fed with Myc at the level of 600 (mg/kg diet). ROS contents in jejunal tissues were significantly decreased dose dependently. Groups fed with higher Myc levels exhibited a remarkable decline in H_2_O_2_ in both muscle and intestinal tissues.

### 3.3. Fecal Oocytes Count, Intestinal Lesion Score and Mortality Percent

The impact of supplementing diets with different Myc levels on oocyte fecal shedding, intestinal lesion score and mortality percent is demonstrated in Table 5. *Eimeria* spp. oocytes were not detected in the feces of the NC group. At 7 dpi, the fecal oocyte count was elevated (*p* < 0.05) in the IC and Myc 200 groups compared with the Myc 400 and 600 groups, and at 14 and 21 dpi, the oocyte count decreased (*p* > 0.05) in the feces of the Myc-supplemented groups in a dose-dependent manner compared with the IC group. Regarding the intestinal lesion score, the IC group displayed (*p* < 0.05) the most severe lesion score in the duodenum, jejunum, ileum and cecum, and the Myc-600-fed group had (*p* < 0.05) a reduced lesion score in all intestinal segments. The highest mortality percent was detected in (*p* < 0.05) the IC group. Notably, the highest (*p* < 0.05) mortality rate was detected at 7 dpi in all infected groups. The maximum (*p* < 0.05) mortality rate was noticed in the infected control group compared with the other groups; moreover, the mortality rate was significantly (*p* < 0.05) decreased with the increase in myricetin level.

### 3.4. Serum Inflammatory and Immune-Related Biomarkers

The impact of supplementing diets with varying levels of Myc on the serum immune-related biomarkers of Ross broiler chickens challenged with *Eimeria* spp. is presented in Table 6. At d 4 pre-infection, the supplementation of the broilers’ diets with Myc improved the birds’ immune-associated parameters via increasing (*p* < 0.05) the levels of IgG and decreasing CRP values, especially at higher doses. At 14 dpi, post-coccidial infection, NO, CRP and MPO were significantly (*p* < 0.05) elevated in the IC group compared to the NC group, and supplementation with Myc decreased (*p* < 0.05) their serum levels, especially at higher doses. Moreover, the highest serum IgG level was detected in the Myc-600-fed group (*p* < 0.05). At 21 dpi, NO, CRP and MPO levels were significantly (*p* < 0.05) decreased in groups supplemented with Myc when compared with the IC group. Moreover, serum IgG levels were significantly (*p* < 0.05) elevated by Myc supplementation, especially at high doses compared to the IC group.

### 3.5. Intestinal and Muscles Antioxidants Gene Expression

**Figure 1:**
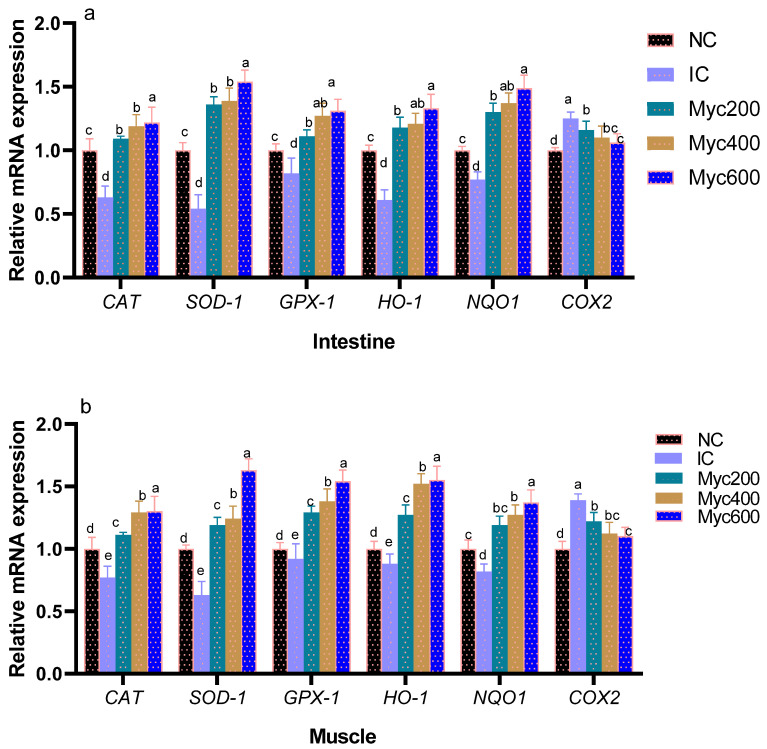
Effect of supplementing diets with varying levels of myricetin on the relative expression of glutathione peroxidase (*GSH*-*Px*), superoxide dismutase (*SOD*), catalase (*CAT*), NAD(P)H dehydrogenase quinone 1 (*NQO1*), heme oxygenase-1 (*HO*-*1*), and cyclooxygenase-2 (*COX-2*) genes in intestinal (**a**) and muscle tissues (**b**) of Ross broiler chickens post infection with *Eimeria* spp. Various letters in columns point to statistical significance (*p* < 0.05). Data are described as means ± SE. NC (negative control): birds fed with basal diet; IC (positive control): birds fed with basal diet and challenged with *Eimeria* spp. at d 14 of age; Myricetin (Myc) 200, 400 and 600: birds fed with basal diet supplemented with Myc at the levels of 200, 400 and 600 mg/kg.

The impact of supplementing diets with varying levels of Myc on the expression of antioxidant-related genes is shown in Figure 1. Intestinal and muscle *CAT*, *SOD*, *GSH-Px*, *HO-1* and *NQO1* gene expression were significantly downregulated in the IC group when compared with the NC group. Moreover, Myc supplementation significantly upregulated (*p* < 0.05) their expression with increasing their dose. In contrast, the expression of COX-2 genes was significantly upregulated in the IC group, unlike the NC group. Interestingly, groups fed with Myc significantly downregulated COX-2 expression (*p* < 0.05) in a dose-dependent manner in both muscles and intestines.

### 3.6. Cytokines and Chemokines Gene Expression

**Figure 2:**
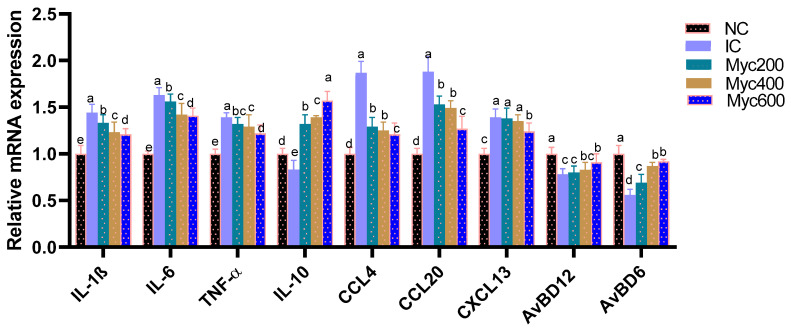
Effect of supplementing diets with varying levels of myricetin on relative expression of interleukin (*IL*)-*6*, *IL*-*1β* and *IL*-*10*, tumor necrosis factor-α (*TNF*-α), (chemokine C–C motif ligand 4, also known as macrophage inflammatory proteins -1β), (*CCL4*), chemokine C–C motif ligand 20, also known as macrophage inflammatory proteins -3 α (*CCL20*) and stromal cell-derived factor-1 (*CXCL13*) Avian β-defensin 6 and 12 (*AvBD6* and *AvBD612*), post infection of Ross broiler chickens with *Eimeria* spp. Various letters in columns point to statistical significance (*p* < 0.05). Data are described as means ± SE. NC (negative control): birds fed with basal diet; IC (positive control): birds fed with basal diet and challenged with *Eimeria* spp. at d 14 of age; Myricetin (Myc) 200, 400 and 600: birds fed with basal diet supplemented with Myc at the levels of 200, 400 and 600 mg/kg.

The impact of supplementing diets with varying levels of Myc on the expression of cytokine- and chemokine-related genes in intestinal tissues is shown in Figure 2. The highest expression of proinflammatory cytokine-related genes as *IL-1β, IL-6* and *TNF-α* was detected (*p* > 0.05) in the IC group, and Myc supplementation significantly (*p* < 0.05) downregulated them in a dose-dependent manner. In contrast, the expression of anti-inflammatory cytokine IL-10 and the AvBD6 and AvBD612 genes were significantly (*p* > 0.05) downregulated in the IC group. Moreover, Myc supplementation prominently (*p* > 0.05) upregulated their levels. Regarding the expression of chemokines involving CCL4, CCL20 and CXCL13, their expression was significantly (*p* > 0.05) upregulated in the IC group in comparison; moreover, higher levels of Myc supplementation significantly (*p* > 0.05) downregulated their expression.

## 4. Discussion

The distribution of the oxidative balance that emerges from oxidative stress, owing to higher free radical production, can trigger cell chain reactions, which in turn results in cell damage or even death. Invasion by the *Eimeria* species can evoke the over production of ROS and free radicals in the host’s cellular immune response and goes beyond the protection capability of the natural antioxidant defense system, which contributes to tissue damage and pathological lesions [45]. Oxidative stress can trigger inflammation that modifies gene expression related to antioxidant status, playing a critical role in the physiological function of the gastro intestinal tract [46]. Antioxidant-enriched diets are among the most substantial dietary factors for poultry, which have unique consequences not only for maintaining their better growth and preventing them from various diseases but also affecting the quality of their products offered for consumers. Plants or their bioactive principles enriched with flavonoids are considered an alternative approach to treat coccidiosis [47]. Herein, dietary myricetin harbors strong antioxidants properties that attenuate the impaired host oxidative equilibrium resulting from coccidian experimental infection. During the starter stage, the dietary inclusion of myricetin in broilers’ diets improved body weight gain and the efficiency of the feed conversion ratio. In accordance, feeding with dietary polyphenol-enriched grape seeds improves the body weight gain and antioxidant status of broiler chicks [48]. In many studies, plant-derived flavonoids have been reported to have an effective role in reducing lipid oxidation, decreasing the pathogenic microbial loads in birds’ intestines and intestinal pH and improving the histomorphology of the intestine, leading to maximizing nutrient absorption and promoting growth performance [49,50]. Myricetin is a plant-derived flavonoid that exhibits many activities in animals’ bodies as a growth promoter with anti-inflammatory, antioxidant and anticancer properties [51]. Before infection, dietary myricetin enhances the immune response and antioxidant status of birds and consequently enhances their performance, including improving their growth rate and FCR. Coccidiosis can induce intestinal oxidative stress that greatly impairs the growth rate and feed efficiency of birds [11]. Remarkably, after *Eimeria* spp. challenges, the body gain of birds was greatly impaired in the IC group that was fed with no additives, and the Myc-supplemented groups, especially at 600 mg/kg, restored these impaired growth performance parameters. Similar results were reported by Bozkurt et al. [52], as the growth performance of broilers and feed conversion ratio worsened after coccidial infection. Moreover, the main clinical signs of coccidial infection are reductions in body weight gain and feed intake with high feed conversion and high economic losses. The alleviated growth performance due to flavonoid supplementation is in agreement with the findings of Wang et al. [47], who reported improved growth performance of experimentally coccidial-infected birds fed with diets supplemented by grape seed extract. The improvement of the birds’ performance may be attributed to the anti-inflammatory effect of flavonoids that reduces the effect of the coccidial destructive effect on the intestine, ameliorating intestinal health status and decreasing diarrhea [53]. Moreover, the anticipated mechanism of action of flavonoids also resulted from their hydroxyl groups, which acted as pro-oxidants that oxidized via ROS inside cell membranes, in turn delaying the bad consequences (lipid oxidation and DNA damage) [54].

In the current study, we assessed the efficacy of myricetin against the severity of coccidian infection via evaluating fecal oocyst counts after infection at 7, 14 and 21 dpi. Coccidian infection exaggerated oocyst excretion per gram of feces after 7 dpi, especially in the infected non supplemented control. The rate of oocyst excretion in feces was observed to decrease as the number of days post infection increased in all groups. Interestingly, the rate of oocyst excretion was much lower in groups supplemented with higher doses of dietary myricetin compared to that of the infected control. In correlation, the intestinal lesion score in all segments of the intestine was reduced with increasing the supplementation levels of myricetin. Phytochemicals as herbal plant extracts or their active substances with antioxidant functions have been developed to be used as anti-parasitic agents, especially coccidiosis such as garlic extract [55], cinnamaldhyde [56], Chinese herbs [57] and a mixture of thyme, oregano and garlic [58]. The effective role of flavonoids and polyphenols in reducing oocyst shedding in the feces of broilers was in accordance with the findings of [16,59], in which it was found that the addition of herbal extract enriched with flavonoids reduces coccidial oocyst shedding in feces simultaneously with decreasing the severity of infection. Moreover, our findings are in accordance with Wang et al. [47], who observed decreased fecal oocysts and intestinal lesion scores with increased levels of grape seed extract in broiler chickens. In line with our findings, Liu et al. [53] reported that chlorogenic acid, which is an antioxidant and anti-inflammatory substance that reduces oocyst count in feces, intestinal lesion score and bloody diarrhea, indicates the inhibition of coccidial infection in broiler chickens. The coccidiocidal or coccidiostatic role of flavonoids could be attributed to interrupting the parasitic life cycle via inhibiting its sporulation [60]. Additionally, the main role of flavonoids and polyphenols as anti-coccidials may be due to its mode of action as anti-inflammatory and antioxidant substances that improve gut health through maintaining mucus secretion, increasing gut epithelial integrity, reducing the colonization of pathogenic microbes and improving local intestinal and body immune defense [61,62].

Dietary polyphenolic supplementation can improve the immune systems of the birds via several ways: binding to the immune cells’ receptors and changing the signaling pathway of the cell, causing the regulation and modulation of the immune response of the host against invasive microorganisms; and enhancing the release of anti-inflammatory cytokines that improve the birds’ resistance against infection [63]. The enhanced immunity that decreased fecal oocyst shedding, intestinal lesions and bloody diarrhea due to myricetin supplementation, from our point of view, is the main cause of the decreased mortality percent compared to the IC group. In accordance, reduction in mortality due to coccidial infection was also observed in broiler chickens supplemented with grape seed extract, which is rich in flavonoids and polyphenol compounds [47]. Moreover, Ageratum conyzoides, enriched with flavonoids at the level of 500–1000 mg/kg, revealed a considerable decrease in the oxidative stress produced by *E. tenella*, improving broiler chickens’ performance and reducing mortality [64].

Under normal management conditions of broiler chickens, the dietary supplementation of polyphenols and flavonoids has a crucial role in protecting birds from oxidative stressors and neutralizing the free radicals produced in body cells such as ROS and reactive nitrogen species (RNS) [65]. Plant-derived polyphenolics compounds have been proven to play an important role in the stimulation of immunity, either through cellular immunity through the modulation of the function of immune cells by binding to immune cells receptors, altering their signaling pathway and stimulating their proliferation [66]; or humoral immunity through the elevation of an antibody titer, increasing lysozyme activity and increasing serum immune globulins [67]. Herein, the role of myricetin (400 and 600 mg/kg) in stimulating the immune status of broiler chickens before infection was clear through boosting IgG and lowering CRP levels.

Parasitic infection such as by *Eimeria* spp. can induce inflammatory responses of the host [53]. In our results, after coccidial challenge on day 14, the NO, CRP and MPO levels were significantly elevated in the IC group compared to the non-infected one, and myricetin addition significantly decreased their levels. *Eimeria* spp. infection has been reported to induce plasma NO levels that may be involved in their pathogenesis, as follows; NO is considered to be a toxic substance to sporulated oocysts [68], and the ingestion of NOS inhibitors increases oocyst output [69]. Also, Yan et al. [68] proved that exogenous NO causes the egress of *E. tenella* sporozite from primary chicken kidney cell cultures before parasite replication. However, the high production of NO by the host cell above the host cell’s tolerance due to coccidian infection can cause tissue damage and cell cytotoxicity, which can induce the inflammation and development of clinical signs such as diarrhea, mortality and intestinal lesions [70]. C-reactive protein, considered an acute inflammatory protein that lowers inflammation levels and is highly produced at the site of inflammation or infection by many cells, such as macrophages, lymphocytes and endothelial cells, is considered potential marker of decreased body inflammation and cells damage [71]. Moreover, CRP plays a crucial role in response to the host’s infection through NO release, phagocytosis, apoptosis and cytokine production, particularly IL-6 and TNF-α [72]. Myeloperoxidase is a pro-inflammatory enzyme generated from neutrophilic granulocytes, and it plays an important role in innate cellular immune responses through its potential effect to injure healthy tissue, thus contributing to disease initiation in poultry [73]. Flavonoid supplementation, such as with curcumin, resveratrol and thymol, have been proven to have an immunostimulatory effect through inhibiting the generation of ROS and NOS by supressing MPO and reducing MPO mRNA expression in neutrophiles [74,75]. Additionally, the hummoral immune response of the host has been reported to be activated through an increased antibody titer, espcially the protective IgG titer, after coccidial infection in laying hens [76]. Our results indicate a high titer of IgG in infected birds supplemented with myricetin, escially at higher dosese. Similarly, Liu et al. [53], who reported an improved antibody titer in *Eimeria* spp. challenged birds, fed birds a diet supplemented with antioxidant chlorogenic acid. Notably, at 21 dpi, the excessive inflammatory response in the IC group subsided in myricetin-supplemented groups, which indicates its potent role against coccidial challenges.

The protective consequence of dietary myricetin against coccidian infection in our study was also achieved through the downregulation of proinflammatory cytokines (*IL-1β*, *IL-6* and *TNF-α*) and chemokines (*CCL4*, *CCL20* and *CXCL13*) and the upregulation of anti-inflammatory cytokines (*IL-10*) and *AvBD6* and *AvBD612* mRNA gene expression. In agreement with our observations, flavonoids such as resveratrol have been proven to have a role in the generation and modulation of cytokines and chemokines in different immune cells [77]. The essential role of proinflammatory cytokines such as interleukin 1 (IL-1), IL-6 and TNF-α have a responsibility in the acute-phase inflammation that is associated with general and metabolic changes [78]. Moreover, these proinflammatory cytokines have a crucial role in modulating the host immune response during infection [79]. Moreover, the anti-inflammatory cytokine IL-10 has been reported to have a role in controlling the host’s immune response by limiting the target cell damage during inflammation [80]. Parasite invasion can use IL-10 to downregulate host immunity and reduce pathogen-damaging inflammatory reactions [81].

Macrophage inflammatory proteins are also known as chemotactic cytokines that comprise CCL4 and CCL20, which play an important role in coordinating the host’s immune responses against infection [82]. Furthermore, CCL4 acts as chemoattractant for important immune cells such as monocytes, macrophages, T-lymphocytes, dendritic cells and natural killer cells [83]. Moreover, CCL4 secretion from neutrophils participates in inflammation by attracting other leukocytic cells to the area of inflammation, resulting in resolving the inflammation by macrophage-mediated cells and developing chronic inflammation [84]. Regarding the CCL20 chemokine, it plays a vital role in the initiation of chronic intestinal inflammation in broiler chickens [85]. CXCL13, which is also recognized as stromal cell-derived factor-1, has a high chemotactic impact on lymphocytes that are involved in inflammatory responses of the host against infections [86]. Moreover, resveratrol was found to improve phagocytes’ killing capability, and the inhibition of TNF-α and NF-kB was found to relieve inflammation in damaged livers with hydrogen peroxide [87]. In the same vein, the immune responses of tilapia have been reported to be improved by the upregulation of *IL-10* and *TGF-β* and the downregulation of *IL-1β*, *IL-8* and *TNF-α* mRNA levels after supplementation with quercetin nano particles [88].

Defensin is an indispensable peptide for the host’s defense mechanism, giving it instant defense against microbial invasion. However, the particular role of defensin proteins in local resistance against the infection of *Eimeria* ssp. has not been well explored [89]. Avian β-defensin 6 and 12 exhibit a chemotactic effect and lipopolysaccharide-neutralizing effect for chicken macrophages. In addition, AvBD12 has been proven to be involved in the induction of murine immature dendritic cell migration to the site of inflammation [90]. Herein, increasing the expression of AvBD6 and 12, following dietary supplementation with Myc, indicates its protective role against coccidia infection.

Additionally, COX-2 is an enzyme whose intermediate, arachidonic acid, undergoes bioconversion to inflammatory prostaglandin with consequential cytokine release [91].

Invasion of host cells with *Eimeria* spp. is known to produce oxidative stress through releasing high amounts of free radicals that play a crucial role in the host’s defense mechanism against parasite infection [92]. The concentration of those free radicals may increase cell tolerance and cause cell cytotoxicity and death, cascading the pathogenesis of the disease. Moreover, the production of this massive amount of ROS and NOS in parasitic diseases can exhaust both low molecular antioxidants such as vitamin A, E and C [70] and metal-dependent antioxidants such as GPX, SOD and CAT [93]. In the current study, it seemed that, after coccidia invasion, higher free radical release and high levels of NO and MDA production were the most important factors impairing the natural antioxidant defense system, which comes in agreement with Georgieva, Koinarski [14].

As evidenced in our study of post coccidia challenge, ROS production decreased, and the expression of the *COX-2* gene in the group that was fed with higher Myc levels was downregulated, which are the main messengers that modify the expression of numerous genes implicated in inflammation [94]. Furthermore, coccidia infection greatly downregulated the expression of antioxidant-related genes such as *GPX*, *SOD*, *CAT*, *HO-1* and *NQO1* in both intestinal and muscle tissues, and the supplementation of strong flavanol compounds such as myricetin alleviated oxidative stress and improved the expression of these antioxidants. Herein, the contents of MDA and ROS and H_2_O_2_ levels in intestinal and breast muscle tissues were significantly reduced after the inclusion of elevated levels of Myc. In contrast, higher T-AOC in intestinal and muscle tissues following supplementation with Myc indicated decreased free radical production and lipid peroxidation. These findings suggest that feeding with myricetin strengthens the oxidative stability of birds via activating antioxidant mediators. The role of plant extracts in protection against coccidiosis may be related to their ability to control the impact on lipid peroxidation in intestinal mucosa and decreasing ROS and NOS production and consequently their destructive effects [95]. The same results were obtained by Idris et al. [96], who described that the inclusion of antioxidant-enriched essential oils can alleviate the oxidative stress caused by the invasion of *Eimeria* spp. In agreement with our results, Tsiouris et al. [16] reported improved antioxidant markers and the alleviation of coccidian infection occurring after the addition of high-polyphenol herbal extract.

## 5. Conclusions

The great benefits of natural antioxidant-rich flavonoids encourage the application of novel ones to tackle the stressors that face the modern poultry industry. Considered together, our findings recommend that the dietary inclusion of Myc can reduce the intestinal sporulation and fecal oocyst shedding of *Eimeria* spp. of infected birds. These beneficial outcomes of Myc could be related to its unique antioxidant and immunomodulatory properties that invoke protection against avian coccidiosis with consequences of superior broiler chicken growth. After dietary supplementation with Myc, its ability to maintain good antioxidant capacity for both intestinal tissues and meat, even after coccidia experimental infection, proves its role as a powerful antioxidant additive. Finally, evaluating the proposed mechanisms beyond Myc’s beneficial effects and modifying the immune and antioxidant responses of birds have prospective future applications in poultry farming.

## Figures and Tables

**Table 1:** Feed ingredients and chemical analysis of the basal diet (as dry matter).

Ingredients g/kg	Starter (0–10 Day)	Grower (11–24 Day)	Finisher (25–42 Day)
Yellow corn grain	57.00	60.60	62.00
Soybean meal, 47.5%	35.00	29.00	25.00
Corn gluten, 60%	2.80	4.50	4.00
Wheat bran	--	--	1.90
Soybean oil	1.20	2.00	3.66
Calcium carbonate	1.00	1.00	0.90
Dicalciumphosphate	1.96	1.90	1.60
Common salt	0.30	0.30	0.30
Premix *	0.30	0.30	0.30
DL-Methionine, 98%	0.18	0.14	0.11
Lysine, Hcl, 78%	0.16	0.16	0.13
Anti-mycotoxin	0.10	0.10	0.10
	Analyzed chemical composition		
Metabolic energy, Kcal/Kg	3000.40	3104.52	3202.02
Crude protein, %	23.02	21.44	19.57
Ether extract, %	3.72	4.60	6.24
Crude fiber, %	2.66	2.55	2.63
Calcium, %	1.01	0.98	0.86
Available phosphorus, %	0.50	0.48	0.41
Lysine, %	1.38	1.22	1.10
Methionine, %	0.56	0.52	0.46

* Vitamin and mineral premix supplied per kg of diet, as follows: Vitamin A, 12,500 IU; Vitamin D3, 2300 IU; Vitamin E, 30 IU; Vitamin K3, 6.00 mg; Vitamin B1, 3.85 mg; Vitamin B2, 6.62 mg; Vitamin B6, 1.6 g; Pantothenic acid, 20 mg; Vitamin B12, 0.5 mg; Niacin, 40 mg; Folic acid, 1.5 mg; Biotin, 0.7 mg; Fe, 55 mg; Mn, 65 mg; Cu, 7 mg; I, 0.9 mg; Co, 1.2 mg; Se, 0.30 mg; Zn, 55 mg; and Choline chloride, 600 mg.

**Table 2:** Primer sequences employed for reverse transcription quantitative polymerase chain reaction assay.

Specificity/Target Gene	Primer Sequence (5′-3′)	Accession No.
*CAT*	F-GGGGAGCTGTTTACTGCAAG	NM_001031215.2
	R-GGGGAGCTGTTTACTGCAAG	
*SOD*	F-GGCAATGTGACTGCAAAGGG	NM_205064.1
	R-CCCCTCTACCCAGGTCATCA	
*GSH-Px*	F-AACCAATTCGGGCACCAG	HM590226
	R-CCGTTCACCTCGCACTTCTC	
*HO-1*	F-AAGAGCCAGGAGAACGGTCA	NM_205344
	R-AAGAGCCAGGAGAACGGTCA	
*NQO1*	F-TCGCCGAGCAGAAGAAGATTGAAG	NM_001277620.1
	R-CGGTGGTGAGTGACAGCATGG	
*COX-2*	F-TGTCCTTTCACTGCTTTCCAT	NM_0,011,67718.1
	R-TTCCATTGCTGTGTTTGAGGT	
*IL-6*	F-AGGACGAGATGTGCAAGAAGTTC	NM_204,628
	R-TTGGGCAGGTTGAGGTTGTT	
*IL-1β*	GCTCTACATGTCGTGTGTGATGAG	NM_204,524
	TGTCGATGTCCCGCATGA	
*TNF-α*	F-CCCCTACCCTGTCCCACAA	XM_046900549.1
	R-ACTGCGGAGGGTTCATTCC	
*CCL4*	F: GCAGTTGTTCTCGCTCTTC	NM_204720.1
	R: GCGCTCCTTCTTTGTGAT	
*CCL20*	F: AGGCAGCGAAGGAGCAC	NM_204438
	R: GCAGAGAAGCCAAAATCAAAC	
*CXCL13*	F: GCCTGTGCCTGGTGCTC	NM_001348657.1
	R: TGCCCCCTTCCCCTAAC	
*AVBD6*	F:GCCCTACTTTTCCAGCCCTATT	NM 001001193.1
	R: GGCCCAGGAATGCAGACA	
*AVBD12*	F:TGTAACCACGACAGGGGATTG	NM 001001607.2
	R: GGGAGTTGGTGACAGAGGTTT	
*GAPDH*	F: GGTGGTGCTAAGCGTGTTA	NM205518
	R: CCCTCCACAATGCCAA	

Catalase (*CAT*), superoxide dismutase (*SOD*), glutathione peroxidase (*GSH*-*Px*), heme oxygenase-1 (*HO*-*1*), NAD(P)H dehydrogenase quinone 1 (*NQO1*), cycloox-ygenase-2 (*COX-2*) and interleukin (*IL*)-*6*, *IL*-*1β* and *IL*-*10*, tumor necrosis factor-α (*TNF*-α), (chemokine C–C motif ligand 4, also known as macrophage inflammatory proteins-1β), (*CCL4*), chemokine C–C motif ligand 20, also known as macrophage inflammatory proteins-3 α (*CCL20*) and stromal cell-derived factor-1 (*CXCL13*) Avian β-defensin 6 and 12 (*AvBD6* and *AvBD12*), glyceraldahyde-3-phosphate dehydrogenase (GAPDH).

**Table 3:** Effect of supplementing diets with varying levels of myricetin on growth performance parameters of broiler Ross chickens.

Myricetin (mg/kg Diet)							
Parameters	NC	IC	Myc 200	Myc 400	Myc 600	*p*-Value	SEM
Starter period (0–10 day)							
Initial BW (g/bird)	44.40	44.40	44.40	44.20	44.40	0.979	0.11
BW (g/bird)	269.20 ^d^	268.80 ^d^	280.60 ^c^	286.60 ^b^	291.00 ^a^	<0.001	1.87
BWG (g/bird)	224.80 ^d^	224.40 ^d^	236.20 ^c^	242.40 ^b^	246.60 ^a^	<0.001	1.88
FI (g/bird)	311.60 ^b^	310.80 ^b^	318.40 ^a^	324.00 ^a^	320.40 ^a^	<0.001	1.24
FCR	1.39 ^a^	1.39 ^a^	1.35 ^b^	1.34 ^b^	1.30 ^c^	<0.001	0.01
Grower period (11–24 day)							
BW (g/bird)	1130.40 ^a^	1065.80 ^d^	1083.40 ^c^	1097.00 ^b^	1127.40 ^a^	<0.001	5.15
BWG (g/bird)	861.20 ^a^	797.00 ^d^	802.80 ^d^	810.40 ^c^	836.40 ^b^	<0.001	4.96
FI (g/bird)	1281.20 ^cd^	1356.40 ^a^	1329.20 ^b^	1286.40 ^c^	1266.40 ^d^	<0.001	7.09
FCR	1.49 ^e^	1.70 ^a^	1.66 ^b^	1.59 ^c^	1.51 ^d^	<0.001	0.02
Finisher period (25–42 day)							
BW (g/bird)	2602.33 ^a^	2145.67 ^d^	2267.33 ^c^	2459.67 ^b^	2496.67 ^b^	<0.001	34.00
BWG (g/bird)	1471.93 ^a^	1079.87 ^d^	1183.93 ^c^	1362.67 ^b^	1369.27 ^b^	<0.001	29.24
FI (g/bird)	2595.73 ^a^	2360.00 ^c^	2342.67 ^c^	2483.67 ^b^	2416.67 ^bc^	<0.001	22.23
FCR	1.76 ^c^	2.19 ^a^	1.98 ^b^	1.82 ^c^	1.76 ^c^	<0.001	0.03
Overall performance (0–42 day)							
Final BW (g/bird)	2602.33 ^a^	2145.67 ^d^	2267.33 ^c^	2459.67 ^b^	2496.67 ^b^	<0.001	34.00
Total BWG (g/bird)	2557.93 ^a^	2101.27 ^d^	2222.93 ^c^	2415.47 ^b^	2452.27 ^b^	<0.001	34.01
Total FI (g/bird)	4188.53 ^a^	4027.20 ^b^	3990.27 ^b^	4093.73 ^ab^	4003.47 ^b^	<0.001	19.29
Overall FCR	1.64 ^d^	1.92 ^a^	1.80 ^b^	1.69 ^c^	1.63 ^d^	<0.001	0.02

BW = body weight; BWG = body weight gain; FI = feed intake; FCR = feed conversion ratio. ^a,b,c,d,e^ Means within a row carrying different superscript letters denote significant differences (*p* < 0.05). NC (negative control): birds fed with basal diet; IC (positive control): birds fed with basal diet and challenged with *Eimeria* spp. at d 14 of age; Myricetin (Myc) 200, 400 and 600: birds fed with basal diet supplemented with Myc at the levels of 200, 400 and 600 mg/kg.

**Table 4:** Effect of supplementing diets with varying levels of myricetin on oxidative/antioxidant biomarkers in muscle and intestinal tissues.

Myricetin (mg/kg Diet)							
Parameters	NC	IC	Myc 200	Myc 400	Myc 600	*p*-Value	SEM
Muscle Tissues							
T-AOC (U/mg of protein)	1.79 ^c^	1.62 ^c^	2.46 ^b^	2.98 ^ab^	3.35 ^a^	<0.001	0.69
MDA (nmol/g tissue)	19.96 ^b^	21.69 ^a^	19.10 ^b^	18.32 ^c^	16.20 ^d^	<0.001	1.29
ROS	63.47 ^b^	86.14 ^a^	64.12 ^b^	63.52 ^b^	57.66 ^c^	<0.001	3.03
H_2_O_2_ (µmoL/g tissue)	2.69 ^b^	3.85 ^a^	2.31 ^c^	2.21 ^cd^	2.01 ^d^	<0.001	0.53
Intestinal Tissues (Jejunum)							
T-AOC (U/mg of protein)	1.13 ^c^	0.63 ^d^	1.26 ^b^	1.29 ^b^	1.39 ^a^	<0.001	0.022
MDA (nmol/g tissue)	15.32 ^b^	21.69 ^a^	13.55 ^c^	12.90 ^d^	12.11 ^e^	<0.001	1.36
ROS (μL/g tissue)	58.17 ^b^	66.10 ^a^	58.18 ^b^	55.12 ^c^	51.60 ^d^	<0.001	5.15
H_2_O_2_ (µmoL/g tissue)	2.23 ^b^	3.99 ^a^	1.96 ^c^	1.78 ^cd^	1.61 ^d^	<0.001	0.50

T-AOC: total antioxidant capacity; MDA: malondialdehyde, ROS: reactive oxygen species, H_2_O_2_: hydrogen peroxide. ^a,b,c,d,e^ Mean values with various letters in the same row are significantly different at *p* < 0.05. Data are described as means ± SE. NC (negative control): birds fed with basal diet; IC (positive control): birds fed with basal diet and challenged with *Eimeria* spp. at d 14 of age; Myricetin (Myc) 200, 400 and 600: birds fed with basal diet supplemented with Myc at the levels of 200, 400 and 600 mg/kg.

**Table 5:** Effect of supplementing diets with varying levels of myricetin on fecal oocyte count, intestinal lesion score and mortality % of broiler Ross chickens.

Myricetin (mg/kg Diet)							
Parameters	NC	IC	Myc 200	Myc 400	Myc 600	*p*-Value	SEM
Fecal oocytes count (×10^3^/g feces)							
7 dpi	ND	355.40 ^a^	352.20 ^a^	340.00 ^b^	339.60 ^b^	<0.001	28.36
14 dpi	ND	227.60 ^a^	212.80 ^b^	169.60 ^c^	135.20 ^d^	<0.001	16.62
21 dpi	ND	74.20 ^a^	54.40 ^b^	44.00 ^c^	34.80 ^d^	<0.001	5.03
Intestinal lesion score 7 dpi							
Duodenal lesion score	ND	3.40 ^a^	3.00 ^b^	2.80 ^b^	2.60 ^c^	<0.001	0.27
Jujenal lesion score	ND	3.80 ^a^	3.60 ^a^	3.20 ^a^	2.20 ^b^	<0.001	0.29
Ileal lesion score	ND	3.80 ^a^	3.40 ^a^	3.40 ^a^	2.00 ^b^	<0.001	0.30
Cecal lesion score	ND	3.60 ^a^	3.20 ^a^	3.20 ^a^	2.20 ^b^	<0.001	0.28
Mortality % overall the experimental period							
Mortality % 7 dpi	-- ^e^	18.00 ^a^	10.00 ^b^	8.80 ^c^	7.40 ^d^	<0.001	1.20
Mortality % 14 dpi	0.80 ^d^	11.00 ^a^	8.00 ^b^	5.00 ^c^	5.00 ^c^	<0.001	0.76
Mortality % 21 dpi	-- ^e^	5.00 ^a^	4.00 ^b^	3.00 ^c^	2.00 ^d^	<0.001	0.45

^a,b,c,d,e^ Means within a row carrying different superscript letters denote significant differences (*p* < 0.05). ND: not detected; dpi: days post infection; NC (negative control): birds fed with basal diet; IC (positive control): birds fed with basal diet and challenged with *Eimeria* spp. at d 14 of age; Myricetin (Myc) 200, 400 and 600: birds fed with basal diet supplemented with Myc at the levels of 200, 400 and 600 mg/kg.

**Table 6:** Effect of supplementing diets with varying levels of myricetin on serum inflammatory and immune-related biomarkers of Ross broiler chickens.

Myricetin (mg/kg Diet)							
Parameters	NC	IC	Myc 200	Myc 400	Myc 600	*p*-Value	SEM
Pre infection (4 days pre coccidian infection)							
NO (µmol/L)	0.64	0.64	0.63	0.60	0.59	<0.07	0.01
CRP (mg/L)	3.51 ^a^	3.51 ^a^	3.32 ^b^	3.01 ^c^	2.73 ^d^	<0.001	0.06
MPO (µmol/L, OD 450 nm)	0.45	0.45	0.42	0.44	0.47	0.013	0.06
IgG (mg/dL)	1.67 ^c^	1.67 ^c^	1.81 ^b^	1.86 ^b^	2.38 ^a^	<0.001	0.06
14 days post infection							
NO (µmol/L)	0.65 ^d^	1.28 ^a^	0.99 ^b^	0.99 ^b^	0.87 ^c^	<0.001	0.04
CRP (mg/L)	3.51 ^d^	10.58 ^a^	8.60 ^b^	8.67 ^b^	6.92 ^c^	<0.001	0.49
MPO (µmol/L, OD 450 nm)	0.46 ^d^	1.67 ^a^	1.50 ^b^	1.49 ^b^	1.25 ^c^	<0.001	0.09
IgG (mg/dL)	1.70 ^d^	5.50 ^c^	6.67 ^b^	6.78 ^b^	7.46 ^a^	<0.001	0.42
21 days post infection							
NO (µmol/L)	0.65 ^e^	1.02 ^a^	0.85 ^b^	0.79 ^c^	0.72 ^d^	<0.001	0.03
CRP (mg/L)	3.49 ^d^	7.16 ^a^	5.46 ^b^	5.45 ^b^	4.58 ^c^	<0.001	0.25
MPO (µmol/L, OD 450 nm)	0.44 ^d^	1.25 ^a^	0.90 ^b^	0.80 ^b^	0.66 ^c^	<0.001	0.06
IgG (mg/dL)	1.66 ^d^	6.40 ^c^	7.80 ^b^	7.71 ^b^	8.64 ^a^	<0.001	0.51

NO: nitric oxide; CRP: c-reactive protein; MPO: myeloperoxidase; IgG: immunoglobulin-G. ^a,b,c,d,e^ Mean values with various letters in the same row are different significantly at *p* < 0.05. Data are described as means ± SE. NC (negative control): birds fed with basal diet; IC (positive control): birds fed with basal diet and challenged with *Eimeria* spp. at d 14 of age; Myricetin (Myc) 200, 400 and 600: birds fed with basal diet supplemented with Myc at the levels of 200, 400 and 600 mg/kg.

## Data Availability

The data presented in this research will be offered upon request to the corresponding author.
